# 

**Published:** 1827-11

**Authors:** 


### MONTHLY LIST OF MEDICAL BOOKS.

Reports of Medical Cases, selected with a View of illustrating the Symptoms and Cure of Diseases by a reference to Morbid Anatomy; embracing Dropsy, Inflammation of the Lungs, Phthisis, and Fever. By RICHARD BRIGHT, M.D. F.R.S. &c. Lecturer on the Practice of Medicine, and one of the Physicians to Guy's Hospital.—4to. with coloured Plates. Loncon, 1827.

A Manual of Comparative Anatomy, translated from the German of J. F. BLUMENBACH, with additional Notes, by WILLIAM LAWRENCE, Esq. F.R.S. Surgeon to St. Bartholomew's Hospital, to Bridewell and Bethlem Hospitals, &c. &c. Second Edition, revised and augmented, by WM. COULSON, Demonstrator of Anatomy at the Medical School, Aldersgate-street, and Member of the Zoological Society.—8vo. London, 1827.

*** The Editor of Blumenbach's valuable work states in his advertisement the circumstances in which it differs from the former edition. It is unquestionably a work of great merit and research, and forms an important addition to the stock of medical literature.

"With respect to the new disposition of the matter, the notes of Blumenbach have been incorporated, wherever it has been practicable to effect such an union, with the text; an arrangement which is sanctioned, in many instances, by the authority of the author himself in the later editions of his work; and which will be found, it is hoped, to contribute not less to the profit than to the convenience of the student. Frequent annotations necessarily divert the attention of the reader from the chain of reasoning, or the detail of facts, which the text may present to him; and there is the less reason for isolating the information contained in the notes of Blumenbach, as it is for the most part strictly relevant to the subject matter of the text. The additional notes of Mr. Lawrence, which, in the edition of 1807, were annexed *en masse* to the end of each chapter, have, in this edition, been printed in a distinct type at the end of each paragraph of the text which they are designed to illustrate.—Of the new matter, part has been introduced by the author in editions of this Manual subsequent to that translated in 1807, and part has been annexed to, or incorporated with, the notes of Mr. Lawrence, by the present editor."

The Lectures of Sir Astley Cooper, Bart. F.R.S. Surgeon to the King, &c. &c. on the Principles and Practice of Surgery; with additional Notes and Cases, by Frederick Tyrrell, Esq. Surgeon to St. Thomas's Hospital, and to the London Ophthalmic Infirmary. Vol. III.—8vo. London, 1827.

A Treatise on those Diseases which are either directly or indirectly connected with Indigestion: comprising a Commentary on the principal Ailments of Children. By David Uwins, M.D. &c.—8vo. London, 1827.

The Dissector, Nos. I. II. and III.

An Essay on Insanity, or Mental Aberration. By Edward Sutleffe.

A Grammatical Introduction to the London Pharmacopœia; containing a short but comprehensive Latin Grammar, Selections, Vocabulary, &c. &c. By S. F. Leach.—18mo. London, 1826.

*** This will be found a very useful little work by those students who have neglected to make themselves acquainted with the Latin Grammar, or who may have forgotten it. It is, in fact, a grammar, founded on the London Pharmacopœia, and appears to be extremely well adapted to its avowed purpose.

### METEOROLOGICAL JOURNAL,

### NOTICES.

*Communications have been received from* Dr. Macartney, Mr. E. G. Ballard, Mr. Jones, Dr. Charles Thomas, Mr. Hamilton, Mr. Raing, *and* Dr. Paul

☞ *It is particularly requested that all Communications relating to the business of the Journal may be sent to the Publisher, not to the Editor.*

